# Editorial: Multi-omic data integration

**DOI:** 10.3389/fcell.2015.00046

**Published:** 2015-07-07

**Authors:** Christine Nardini, Jennifer Dent, Paolo Tieri

**Affiliations:** ^1^LazzariBologna, Italy; ^2^Group of Clinical Genomic Networks, Key Laboratory of Computational Biology, CAS-MPG Partner Institute for Computational Biology, Shanghai Institutes for Biological SciencesShanghai, China; ^3^QuintilesReading, UK; ^4^Consiglio Nazionale delle Ricerche, Istituto per le Applicazioni del CalcoloRome, Italy

**Keywords:** multi-omics, multi-omic data integration, integration, systems biology, network analysis

As researchers involved in molecular biology, we are witnessing tremendous paradigm changes in a time frame that becomes shorter and shorter. The epoch-making notion, originally put forward by the central dogma of biology (Crick, 1970), that there is a unidirectional process and a privileged level (genetic) of causality at which biological functions are determined, has already long and strongly been challenged. It is in fact well recognised that multi-level causality with feedback cycles among all former and newly identified biochemical levels (including small RNAs, epigenomic changes) is a fundamental attribute of biological systems (Noble, 2012).

Yet, the focus shift from single reactions to transcriptomics, promoted by microarray first and sequencers now, is already challenged by a novel, pressing offer from fast evolving technologies. Indeed, the possibility to have a *omic* view on virtually all molecular layers (genomes, metagenomes, transcriptomes, proteomes, epigenomes) pushes to integrate the study of systems at yet another level of complexity, a run harmed, and not negligibly, by the difficulties in formatting, storing, and reusing the deluge of data encompassing every level of biological organization.

In such a complex background, it is growingly acknowledged that tools and theoretical frameworks that could help in combining and giving account for both the multi-level causation scheme and the burden of data are still underdeveloped (Witzany and Baluska, 2012).

From these considerations, a novel, pressing request arises to design methodologies, approaches and frameworks that allow for these data to be interpreted as a whole, i.e., as intertwined molecular signatures containing genes, proteins, mRNAs, and miRNAs, but also epigenomic characterizations, as well as correlations with microbiomes' compositions, just to name the major, able to capture the inter-layers connections and the complexity of phenotypes. This request is seconded by demands and concerns about the storage and reusability of much of such different omic data. Indeed, although publicly and freely available, these data often lie in databases and repositories underutilized or not used at all. Issues coming from lack of standardization and shared biological identities are also well known to represent a hurdle for data reuse (Tieri and Nardini, 2013; Chowdhury and Sarkar, 2015).

The “Multi-Omic Data Integration” Research Topic is in our intention a dedicated forum to collect efforts that help in defining this emerging field, aimed to the integration of data, analyses and approaches from, and for multiple omics.

The articles here collected address these questions from a number of perspectives that we summarize as *experimental, network based*, and *methodological.* In the first category the authors extract and analyse different types of high-throughput data (epitomics, localisomics, transcriptomics, lipidomics) from different locations on model organisms [*Arabidopsis thaliana* (Wilson et al., 2015) and *rhesus macaques* (Lee et al., 2014)] to understand a complex biological question (roots' growth and response to anti-malarial drugs) that could not be addressed with single-omic approaches.

We transition from these approaches to more theoretical ones via the usage of graphs. Networks offer a complete, intuitive, versatile, and powerful approach to the representation of complex systems (genomics, epigenomics, transcriptomics, metabolomics, host-microbiome interface, diseases' phenomics) which is here exploited to represent the multifaceted aspects of complex autoimmune diseases (*rheumatoid arthritis*, Tieri et al., 2014) in order to evaluate complex side effects of old and novel therapies; to identify disease molecules that can be both effective therapeutic targets relevant progression markers with application to *diabetic nephropathy* (Heinzel et al., 2014); to stratify patients with comorbidities (Moni and Lio, 2015).

Methodological approaches point with a novel emphasis at the importance of molecules' spatial localization in the omic context. From polysome and ribosome profiling, RNA, and miRNA binding sites annotation and standardization (Dassi and Quattrone, 2014), to networks including 3D molecules' proximity thanks to Chromosome Conformation Capture (3C) and its omic version Hi-C (Merelli et al., 2015), spatial representation contributes with an important layer of information in this added multi-omic complexity.

Beyond spatial organization, temporal progression and causal inference are discussed to model the heterogeneity of CD4^+^ T cells and their complex immune responses (Carbo et al., 2014), and to predict gene networks based on ChIP-seq and RNA-seq integration (Angelini and Costa, 2014).

Finally, meta analyses of genomes, be it for the exploration of microbiomes' compositions or disease genome-wide association studies (GWAS) still benefit from discussion in this research topic, on one side for the need of standardization of the workflow (Ladoukakis et al., 2014) in a relatively novel research area (omic microbiology) and on the other side to compensate with multi-omic layers to the limited statistical power and reproducibility of GWAS (Lin et al., 2014).

This collection is the tip of an iceberg that continues to grow and to evolve in multiple directions. From the continuously improving efficiency of existing high-throughput platforms that imply easier, cheaper and more frequent spatio-temporal sampling, to the input of novel technologies that will offer omic views on novel types of data (phenotypes, tissues, 3D proteins etc., all entailing the production and approval of dedicated standards for data storage) we are only at the beginning of almost endless possibilities of data integration.

However, to avoid getting lost in the sea of data, efficient algorithms as well as biologically meaningful directions in which to integrate information will be of importance. This will imply not only the implementation of powerful tools to give answers, but also the design of careful approaches to form questions.

We hope and foresee that these needs will foster the collaboration between biologists, medical doctors, statisticians, and computer scientists further, transforming the residual perception of this forced cooperation from a burden to a resource. The impact of completing this other type of integration among scientific expertise is difficult to predict at large, but can easily be assumed as a necessary and crucial starting point for the effective implementation of personalized medicine, where patients' and health practitioners' needs are translated into technology and report on systemic markers, offering patients the possibility to be treated as a whole and not as a mere assemblage of parts to be “adjusted.”

## Funding

This project is partialy funded by MoST international cooperation program no. 2013DFA30790, NSFC no. 31070748, EC FP7-PEOPLE-2011-IRSES program, project 294935 “KEPAMOD,” and CAS fellow grant no. 2011Y1SA04.

### Conflict of interest statement

The authors declare that the research was conducted in the absence of any commercial or financial relationships that could be construed as a potential conflict of interest.
